# Biomimetic Receptors and Sensors

**DOI:** 10.3390/s141222525

**Published:** 2014-11-27

**Authors:** Franz L. Dickert

**Affiliations:** Department of Analytical Chemistry, University of Vienna, Waehringer. Str. 38, A-1090 Vienna, Austria; E-Mail: Franz.Dickert@univie.ac.at

**Keywords:** chemical and biochemical recognition, molecular imprinting, artificial antibodies, self-assembled layers, transducers

## Abstract

In biomimetics, living systems are imitated to develop receptors for ions, molecules and bioparticles. The most pertinent idea is self-organization in analogy to evolution in nature, which created the key-lock principle. Today, modern science has been developing host-guest chemistry, a strategy of supramolecular chemistry for designing interactions of analytes with synthetic receptors. This can be realized, e.g., by self-assembled monolayers (SAMs) or molecular imprinting. The strategies are used for solid phase extraction (SPE), but preferably in developing recognition layers of chemical sensors.

## 1. Introduction

Molecular Recognition has been the dominant challenge of chemistry over the last decades. According to J.-M. Lehn, tackling this challenge can lead to biomimetic receptors realized by supramolecular chemistry—molecular systems based on interactions between molecules and ions. In this way, innovative receptors can be designed mimicking biological analogues, such as antibodies and enzymes. These phenomena can be understood according to host—guest chemistry. Synthetic crown ethers, e.g., as synthesized by C.J. Pedersen, show a high selectivity to potassium ions, in analogy to the cyclic antibiotic valinomycin. Template-directed syntheses are often used to create synthetic receptors using biomimetic strategies. These ideas are further developed by molecular imprinting, embedding these receptors created by template-directed synthesis into a robust polymer. Furthermore, these processes are based on self-organisation without time-consuming synthesis. These biomimetic coatings can selectively bind both ions, neutral molecules and complex bioanalytes. Solid phase extraction (SPE) leads to cleaning and selective enrichment of analytes. These materials are favorably used as sensitive layers for sensors combined with a variety of transducer principles, such as optical, electrochemical, and mass‐sensitive detection. Analytes are widely available from the environment, and applications are obvious for environmental challenges, bio/medical and process engineering monitoring. The size of analytes cover a wide range, from sub-nano-metre for molecules to micro-metre for biological particles.

## 2. Summary of the Special Issue

### 2.1. This Special Issue Starts with Molecules as Analytes

Imprinted layers are used to detect small molecules as histamine, serotonin and L-nicotine in solution [1]. Histamine and serotonin are neurotransmitters, which are important for physiological phenomena. Thus, the effect of size, chemical structure and chemical and physical properties, respectively, for the recognition process were tested. A measuring cell was developed with a volume down to 1.0 μL, which is beneficial for biological samples. A very cost effective method, heat-transfer method, is performed for detecting analytes. This measuring cell makes it possible to measure four samples simultaneously. The quadrants of the flow cell can be equipped with different (molecularly imprinted polymers) MIPs, which reduces measurement time. The MIPs were synthesized by a mixture of monomers and crosslinkers such as methacrylic acid, acrylic acid, acrylamide and ethylene glycol dimethacrylate. The polymerisation is started by the initiator azobisiso-butyronitrile. The MIP particles were transferred by a polydimethylsiloxane stamp to a poly-phenylene-vinylene adhesion layer. The sensor effect results from temperature changes of the analyte being included by the MIP. This effect is measured by a miniature thermocouple. Thus, L-nicotine detection was possible in the concentration range from 0.1 μM to 100 μM. The coating with MIPs and NIPs allows to compensate for non-specific effects. MIPs prepared by printing with histamine and serotonin demonstrate selective detection of the analytes of interest. The described methodology is capable to distinguish between analytes in spite of their chemical similarities.

As in the previous paper [1], the heat‐transfer method was also applied in Reference [2] for sensor detection. The sensitivity for monitoring L‐nicotine in aqueous phase can be greatly improved down to 0.35 nM. This was possible by optimizing the parameters of the proportional-integral-derivative (PID) controller feedback loop. Thus, the noise of the equipment was appreciably reduced.

Mass-sensitive sensors are universally applicable for monitoring analytes under ambient conditions [3]. The surface acoustic wave (SAW) oscillator can be operated at higher frequencies, which results in enhanced sensitivity according to the famous Sauerbrey equation. In this paper a delay line working at 300 MHz was designed. Differential measurements via a mixer between two channels makes it possible to compensate for interfering contributions stemming from temperature fluctuations or other influences. Thus, very thin layers can be applied to the delay area of the SAW, which leads to appreciable responses via adsorption phenomena. In this case, SAMs in a height of 2 nm were linked by sulfur atoms to the SAW gold layer. Furthermore, a supramolecular strategy was used by integrating into this monolayer cyclodextrin molecular hollows. The surface assembling of the functionalised SAMs was characterized by AFM. The organophosphorus analyte can easily be detected below 1 mg/m^3^. Furthermore, the rise and recovery times were approximately only one minute. The imprinting process leads to favorable cross sensitivities to other organic vapors.

Polycyclic aromatic hydrocarbons (PAHs) detection at a very minute level is an analytical challenge concerning environmental chemistry [4]. These substances can be recognized both by artificial antibodies and biological systems. This paper uses both strategies to perform the selective analyte detection. Molecularly imprinted polymers are widely used in robust enrichment procedures. After a selective inclusion of the PAH benzo[a]pyrene (B[a]P) the elution was performed with dichloromethane. The material for molecularly imprinted polymer‐based solid phase extraction (MISPE) was synthesized by a copolymer of 4-vinylpyridine/divinylbenzene. The recognition sites were generated by non-covalent imprinting with the template mixture of pyrene and phenanthrene. Thus, the analyte can be isolated from a complex matrix of vegetable oils. The solvent n-hexane was most suitable for B[a]P inclusion by the MIP column. Up to 95% of B[a]P was extracted by this MISPE procedure. The binding and re-binding of B[a]P can be quantitatively followed by fluorescence spectroscopy. The detection of B[a]P was performed by an ELISA test. Different oil samples with a variable fatty acid contents has proven to be a suitable matrix for calibration. The MISPE‐ELISA strategy is an excellent example of molecular recognition with different strategies. Thus, the quality of edible oils can be guaranteed according to the MRL value of 2 μg/kg of B[a]P with the described MISPE‐ELISA procedure.

According to paper [5], the development of a sweetness sensor is a typical task for molecular recognition. Layers consisting of lipid/polymer (PVC) membranes with plasticizers are suitable for this purpose. The molecules tested are intercalated into the membrane. Hydrophobic interactions between the lipid/polymer membrane and these tastants leads to potentiometric responses as sensor signals. Thus, aspartame, an important sweetener can be detected by the designed sensor. This idea was already commercialized.

Imprinting can also be used for selective detection of Tamoxifen [6], which is an estrogen‐receptor modulator, applied in breast cancer therapy. Another analytical challenge is its detection when used as performance-enhancing drug by athletes in sports. The strategy of detection is based on modified electrodes. The selectivity of this electroanalytical method is based on MIPs, which enrich the analyte at the electrode surface. It has to be emphasized that this electrochemical method is a relative inexpensive measuring technique. Glassy carbon disk electrodes were modified by an electropolymerisation of a mixture of o‐phenylenediamine dihydrochloride and resorcinol under the addition of the template. The electrode shows no reduction of ferricyanide under this condition. The removal of the template leads to an increase in reduction current, which is diminished by the inclusion of the analyte. The imprinted polymer is highly selective towards targeted analyte in spite of only a minor variation in the template molecule. This strategy yields very sensitive sensor responses over a wide concentration range from 1 to 100 nM.

The following different topics are discussed in an excellent review article [7] concerning the development of biomimetic sensors for a large variety of analytes.

#### Molecularly Imprinted Polymers

The biorecognition property of enzymes, antibodies, cells, animal or plant tissues can be imitated by following an innovative technique named as molecular imprinting. Imprinted materials are robust materials that can be synthesized without time consuming efforts. The imprinting process can be performed both with organic and inorganic ingredients, but preferably via organic polymers. A template, monomers and crosslinkers are necessary for this process. The polymerization can be started by changing temperature, UV irradiation, or using an initiator. An appropriate solvent can act as porogen, which will favor diffusion processes. Two types of molecular imprinted can be performed, covalent and non-covalent binding of the template.

#### Molecularly Imprinted Membranes

An application of imprinting is the development of imprinted membranes, which are selective barrier between two neighboring phases. Membranes were developed which showed recognition properties towards a great deal of analytes, such as herbicides, drugs, and enantioselective permeation.

#### Biosensors

Biosensors are very important tools for detecting chemical and biological compounds in clinical environmental and food monitoring process. Biosensors comprise a biological recognition element and a transducer for generating an electrical signal.

#### MIMs-Based Bio-Mimetic Sensors

Biological receptors exhibit high selectivity but lack behind artificial ones due to their poor stability, interferences to ambient environmental influences and difficulties to integrate them in devices. These problems can be reduced by employing imprinted materials, especially by using imprinted polymer particles for enhanced sensitivity. Low-weight organic molecules can be detected by following this strategy to detect contaminants in water and food. The membrane enriched pesticides, haloacetic acids, antibiotics, persistent contaminants, drugs and bioactive molecules with a variety of transducer methods. Potentiometric and amperometric sensors are most easily realized.

### 2.2. The Second Part Deals with Bioanalytes up to Cells

This excellent review article [8] describes various sensor applications of metal oxides in detecting very complex biological analytes. Metal oxides are mostly used for gas phase monitoring in respect to reducing and oxidizing gases, resulting in conductometric effects.

In this paper, however, a potentiometric biosensor for the detection of L-lactic acid was described via lactate oxidase immobilized on ZnO. A wide concentration range with a fast response time was accessible. Generally, such type of sensors can be designed by applying some biological recognition systems to the metal oxide surface. The interaction of biological coating with the analyte leads to some electron transfer reactions, which cause changes in physical properties of the metal oxides. The penicillinase enzyme immobilized on this type of support allows the detection of penicillin. Glucose-, or cholesterol oxidase linked to metal oxide nanostructures leads to monitoring of glucose and cholesterol, respectively. Another general detection procedure is based on antibodies such as immunoglobulin G. Thin films of IrO_2_ or Ti/TiO_2_ can also be used for this purpose. Even optical detection via SPR can be applied. Thus, C-reactive protein can be analyzed which is of high importance in clinical analytics since it indicates inflammations in the body. Metal oxides, such as ZnO, CuO nanoflowers, nickel oxides coated with functionalized polymers, are ion sensitive electrodes for thallium (I,), Cd(II) and Zn(II), respectively. A wide dynamic range for detection, accompanied by a sufficient selectivity can be realized.

Molecular imprinting is an innovative strategy to generate synthetic receptors for bioanalytes [9]. In this case molecular hollows were designed suitable for lysozyme inclusion. This enrichment can be monitored by fluorescence spectroscopy. Functionalized multi-walled carbon nanotubes (MWCNTs) are highly promising supporting materials for MIPs, which ensures a stable binding of the sensitive coating to the MWCNTs. In this way a porous phase is created which guarantees quick inclusion of the bioanalyte and its release. The polymer was prepared in aqueous solution of acrylamide and *N*,*N*-methylenebisacrylamide as crosslinker with an addition of the template lysozyme. The polymerization was initiated by ammonium persulfate. The sensor materials were modified by linking to the fluorescence label fluorescein isothiocyanate. Incorporation of lysozyme leads to fluorescence quenching. Stern-Volmer plots were used for a quantitative evaluation of the analytes by the MIPs. The proteins cytochrome c, hemoglobin and bovine serum albumin showed only minor interference in spite of their similar size.

The deoxyribonuclease (DNase) enzyme catalyzes hydrolytic cleavage of phosphodiester linkages in the DNA backbone, thus degrading DNA [10]. A variety of deoxyribonucleases are known with different specificities and biological functions. Ribonuclease (RNase) catalyzes the cleavage of RNA into smaller fragments. DNase can also be used as a marker of diseases, e.g., it causes several autoimmune diseases. It can be detected after fragmentation by gel electrophoresis. Furthermore, a spectroscopic monitoring is possible via the absorbance of nucleotide bases. A very sensitive strategy is realized by fluorescence, e.g., applying graphene oxide or the innovative, so called, FRET method.

RNase can also be electrochemically detected involving ferrocenyl probes. A highly sensitive method for detection of RNase is immobilizing on magnetic beads. Guanine bases can analyzed by stripping chronopotentiometry. The application of nano particles is highly suitable for sensitive detection of the target analyte. The most analytical methods can also be used in the presence of interfering compounds.

Human hematopoietic stem and progenitor cells (HSPCs) are decisive in treating many hematological malignancies [11]. The HSPCs transplantation can generate blood cells in cancer patients with depleted immune cells. Mutagenesis, however, seems also convert HSPCs to leukemic stem cells. Thus, a reliable enrichment of HSPCs by innovative analytical strategies should lead to improvements in transplantation therapy and a better understanding of leukemia. A biomimetic strategy was used for HSPC isolation to simulate the vascular environment caused by trauma. This can be evaluated by interaction with selectins, which are transmembrane glycoproteins. For this purpose microtubes were coated with L-selectin and perfusion of the analyte was studied. Furthermore, the variation of pH is highly important for this process. L-selectin shows conformational changes due to a variation of pH as shown by dynamic light scattering, Which results in a decreased cell rolling velocity and its rolling flux. The described biomimetic sensor technique results in a rapid and simple isolation cell strategy.

Keratinocyte cells in epidermis act as a barrier against environmental threat such as pathogens, or chemical and physical influences, respectively [12]. In case of pathogen invasion, keratinocytes will generate mediators, which attract leukocytes to this site and defend the body against invaders. Reactive oxygen species can significantly damage cell structure. In principal, the characterization of these effects can be performed by direct detection of ROS, damaged biomolecules, or concentration of antioxidant compounds. An optical sensor based on human keratinocytes (HaCaT cells), including the green fluorescent protein (GFP) with the HSP70B promoter was used as a detection strategy. The response of HaCaT sensor cells to 25 μM cadmium chloride leads to an increase in total glutathione. The oxidative stress activates routes for enzyme formation, which is responsible for glutathione biosynthesis. Furthermore, extracts from *Arnica montana* flowers, which acts as a skin irritant was tested. Significant responses of HaCaT cells to treatments with *Arnica montana* extracts were observed. Pronounced effects are given by its dominant helenalin ingredient. These investigations can be extended to a large variety of inorganic and organic chemicals. It is possible to integrate these detection systems in microfluidic devices.
